# Performance Evaluation of Boiling Chamber With Enhanced Boiling and Condensing Surfaces for Efficient Warm Water Operation

**DOI:** 10.1115/1.4070962

**Published:** 2026-04-01

**Authors:** Nooruldeen Essam Mustafa, Satish G. Kandlikar

**Affiliations:** Department of Mechanical Engineering, Rochester Institute of Technology, Rochester, NY 14623; Department of Mechanical Engineering, Rochester Institute of Technology, 76 Lomb Memorial Drive, Rochester, NY 14623; Rochester Institute of Technology

**Keywords:** boiling chamber, 1.5 U server, microchannel substrate, plain substrate, data center cooling, liquid cooling, critical heat flux, high heat flux dissipation, air-cooled data centers, PUE

## Abstract

Boiling and condensation are integrated within a boiling chamber to cool a heating surface, such as a computer substrate, with cooling water. A finned tube condenser with a total surface area of 48,440 mm^2^ is used, and the effect of different fill ratios (40% and 80%) is studied. The results indicate that the enhanced finned tube condenser performs exceptionally well in improving the overall boiling chamber performance. The enhanced boiling chamber is able to dissipate high heat fluxes from the computer substrates within the operating temperature limits of the substrates with warm water cooling. This enables use of higher coolant inlet temperatures enabling the use of dry cooling towers and reducing water consumption in a data center. With a fill ratio of 40% and coolant inlet temperature of 40 °C, the boiling chamber dissipated a heat flux of 178 W/cm^2^ at a surface temperature of 84 °C. With a lower coolant inlet temperature and a fill ratio of 80%, a heat flux of 177 W/cm^2^ was dissipated with a 20 °C coolant inlet temperature, resulting in the surface temperature of 79 °C. This shows the boiling chamber's ability to outperform even at elevated coolant temperatures. Further, the compact design of the boiling chamber is well-suited for its implementation in data center cooling.

## 1 Introduction

Data center workloads have increased exponentially due to cloud computing, artificial intelligence, and massive data storage demands [1]. Hardware reliability critically depends on operating temperature; insufficient cooling can lead to hot spots, accelerating component degradation, and risking premature failure [2,3]. Water consumption in data centers, both direct through evaporative cooling and indirect through electricity generation, poses notable concerns [4]. Efficient thermal management, therefore, reduces operating expenses and lowers the ecological footprint [5]. Most existing facilities rely on air-cooled systems that circulate chilled air through raised-floor plenums. However, air has low thermal conductivity and specific heat, leading to large temperature gradients and poor heat removal from high-power components [6]. Localized hot spots force operators to lower air temperatures, increasing chiller power while overcooling other equipment. Refrigeration systems may need to operate year-round, even in cold climates, because heat is removed indirectly via room air rather than directly from the heat-generating device [7]. Cooling infrastructure accounts for a substantial portion of energy costs, and servers consume 80% of peak power even at 20% utilization [8]. These limitations underscore the need for direct liquid cooling approaches that can efficiently remove high heat fluxes while reducing both energy and water footprints.

Several thermosyphon and loop thermosyphon studies for electronics and server cooling demonstrate the existence of an optimal filling ratio at which thermal resistance is minimized, and operation is most stable. Chang et al. [9] designed a closed-loop thermosyphon. They measured overall and component thermal resistances as a function of working fluid charge, finding optimal performance at approximately 40–60% fill, with the lowest junction-to-ambient resistance near 40%. Undercharging led to intermittent boiling and drying out, whereas overcharging caused condenser flooding and increased pressure drop. Samba et al. [10] and Chehade et al. [11] similarly identified narrow optimal volumetric fill ranges, approximately 9–10% for a CO_2_ loop and 7–10% for a water loop, outside of which vapor starvation at low fill or excessive liquid inventory at high fill degraded performance and stability. Ghaffari et al. [12] investigated a dielectric closed-loop thermosyphon for cooling a central processing unit (CPU) using Novec 7000. They found the best heater to air resistance and maximum power of 315 W at surface temperatures below 80 °C, corresponding to a heat flux of 49 W per square centimeter at a 42% filling ratio, with earlier dry-out at 32% and increased condenser resistance at 52%. Vincent et al. showed that advanced thermosyphon heatsinks for high-power CPUs exhibit a distinct optimal charge, with thermal resistance increasing when the filling ratio deviates by approximately ±10 to 15% due to evaporator dryout or condenser flooding [13]. Minazzo et al. Reported a compact thermosyphon system for an edge microdatacenter with a broad minimum-resistance plateau near a high filling ratio of approximately 75% of the internal volume. In contrast, lower fills produced temperature oscillations, and higher fills caused pressure rise and flooding [14].

Recent studies have investigated strategies to reduce data-center energy and water consumption. Ndukaife and Nnanna showed that hybrid evaporative cooling can serve as a standalone cooling approach when humidity is properly controlled, although water consumption increases nonlinearly with operating time [4]. In air-cooled systems, increasing the allowable rack inlet temperature improves chiller efficiency and reduces cooling power and water usage. Optimizing server utilization, implementing free-cooling economizers, and adopting liquid cooling where appropriate can substantially reduce operational energy and water costs [15]. However, as power densities continue to rise, device-level cooling solutions are required to manage the intense heat generated by modern CPUs and graphic processing unit (GPUs). Vapor chambers and thermosiphons are widely used two-phase devices for electronics cooling. A vapor chamber is a sealed heat spreader that relies on evaporation, vapor transport, condensation, and capillary return within a wick structure to achieve uniform surface temperatures and high effective thermal conductivity [2,16]. Their performance is ultimately limited by capillary constraints, orientation effects, and dry-out at very high heat fluxes. A thermosiphon is a closed-loop system in which the working fluid circulates by buoyancy-driven natural convection [3].

Shukla and Kandlikar developed a subcooled boiling chamber that combines subcooled boiling and submerged condensation to remove high heat fluxes from electronic devices [17]. The chamber employs microstructured surfaces to initiate boiling while maintaining the bulk coolant below its saturation temperature. The boiling chamber was able to dissipate over 1.1 kW of heat from a simulated CPU while keeping the surface temperature below 92 °C, achieving a thermal resistance of less than 0.1 K/W. By combining subcooled boiling with submerged condensation, the design removes heat efficiently and stabilizes the boiling process. Subcooled boiling occurs when the bulk liquid temperature remains below the saturation temperature while the wall temperature exceeds it [18]. This creates vapor bubbles at the heated surface that collapse as they move into the cooler bulk liquid. Boiling offers much higher heat transfer coefficients than single-phase convection because the latent heat of vaporization absorbs large amounts of energy while keeping the surface temperature low. The high heat-transfer capability of boiling enables the removal of significant heat fluxes over small surface areas, providing a path to dissipate high heat from electronic devices while maintaining safe temperatures [7]. However, excessive heat flux can trigger dry-out, leading to a sudden rise in surface temperature. Submerged condensation refers to the condensation of vapor bubbles within a liquid environment rather than on a cold solid surface [19]. When vapor generated during subcooled boiling rises into cooler regions of the liquid, it condenses within the liquid volume, releasing latent heat directly to the surrounding fluid. The boiling chamber's performance is limited by the performance of the individual condenser and boiling surface, and this study focuses on improving overall performance.

## 2 Objective of This Study

The objective of this study is to enhance condenser performance by incorporating finned tube design and to investigate performance improvement with different fill ratios. The specific objectives are as follows:

1. Design a finned tube condenser with a large surface area in a boiling chamber configuration.
2. Evaluate system performance with plain and microchannel substrates for different fill ratios ranging from 40% to 80% of the boiling liquid in the boiling chamber.
3. Evaluate system performance with different coolant inlet temperatures between 20 °C and 50 °C.

## 3 Research Method

### 3.1 Test Substrates.

**Fig. 1:**
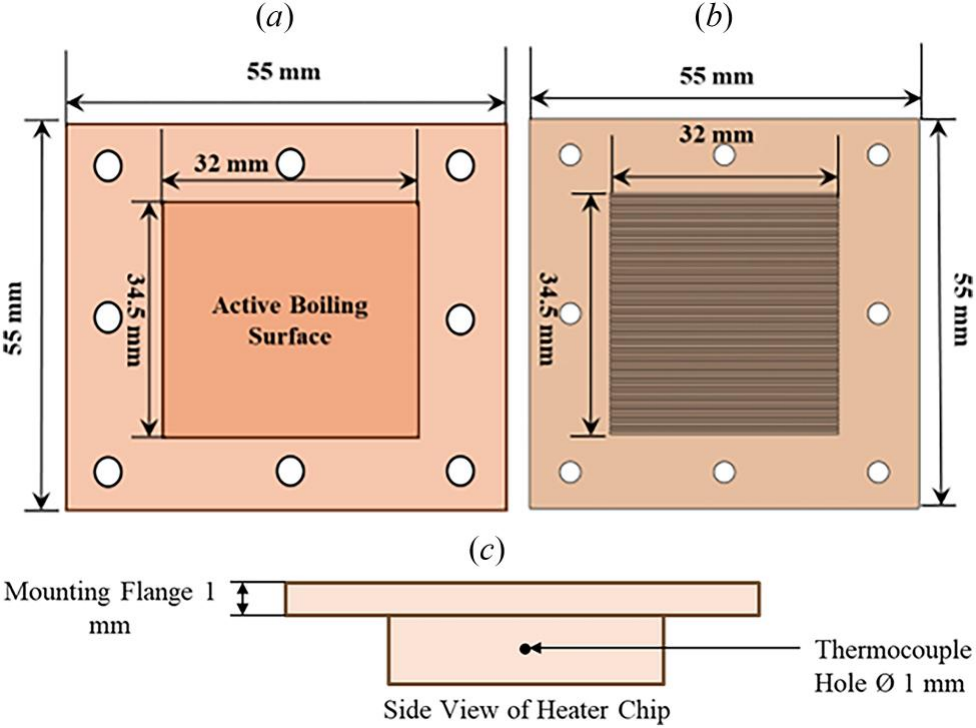
(*a*) Schematic of the plain copper substrate showing the central 32 mm × 34.5 mm active boiling surface. (*b*) Schematic of the microchannel copper substrate with parallel microchannels occupying the same active boiling area. Shown below is a side view of the heater substrate depicting the thickness of the boiling surface. Adapted from Shukla and Kandlikar [11].

The test heater substrates were fabricated from copper alloy 101, which possesses a thermal conductivity of 391 W/(m·K). A schematic of both the plain and microchannel copper substrates is presented in Fig. 1. Each substrate features a central active boiling area of 34.5 mm × 32 mm with an average surface roughness of 2.5 *μ*m, which is at the same level as the mounting flange, facilitating efficient heat transfer. The substrates used in this study are 1 mm thick, with overall dimensions of 55 mm × 55 mm × 1 mm.

A 500 *μ*m diameter T4 thermocouple probe is inserted through a hole drilled on one of the 32 mm wide edges, allowing for the measurement of internal substrate temperature to estimate surface temperature under varying heat fluxes. The boiling area on the top surface is delineated with Kapton tape for visual reference during testing.

As shown in Fig. 1(*b*), the microchannel substrate features parallel channels (500 *μ*m wide × 400 *μ*m deep) separated by 200 *μ*m fins. This designed geometry significantly enhances boiling heat transfer and phase-change efficiency by expanding surface area and strategically controlling nucleation sites.

### 3.2 Experimental Setup.

**Fig. 2:**
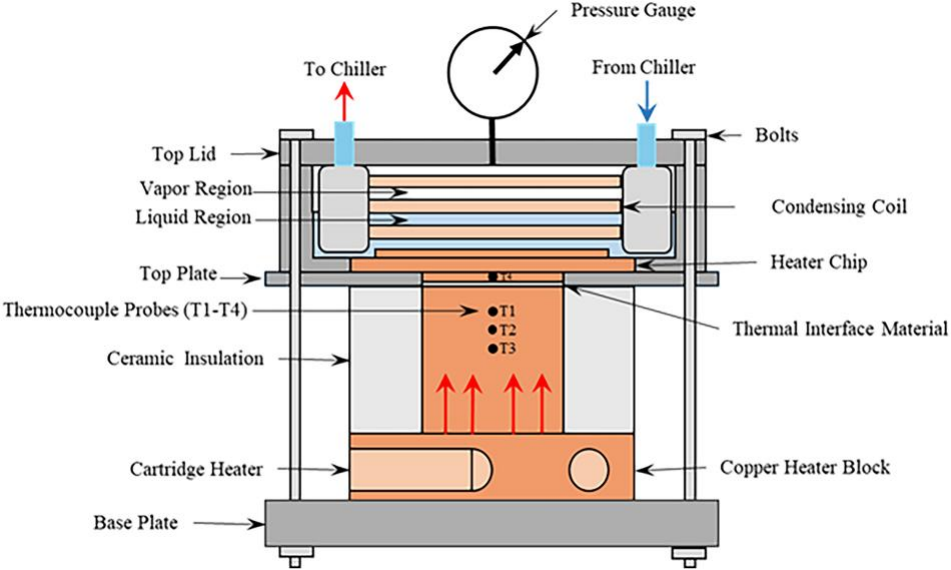
Boiling chamber experimental schematic, highlighting the key components. Adapted from Shukla and Kandlikar [11].

The experimental arrangement, shown in Fig. 2, centers around a custom–built boiling chamber coupled to a data–acquisition system for accurate monitoring of temperature and heat flux. The chamber itself is a 1.5 U unit measuring 70 mm by 85 mm by 30 mm internally, rising to 42 mm overall height when the lid is bolted down with six socket–cap screws. To ensure safe and repeatable operation, the chamber is fitted with a pressure gauge (dual-scale pressure and vacuum gauge with steel case, 2 in. dial diameter, 1/4 in. National Pipe Thread (NPT) bottom connection) for continuous vapor–pressure readings and a needle valve that allows the working fluid to be charged and vented. Two bulkhead fittings connect the chamber's internal condenser coil to an external chiller loop.

**Fig. 3:**
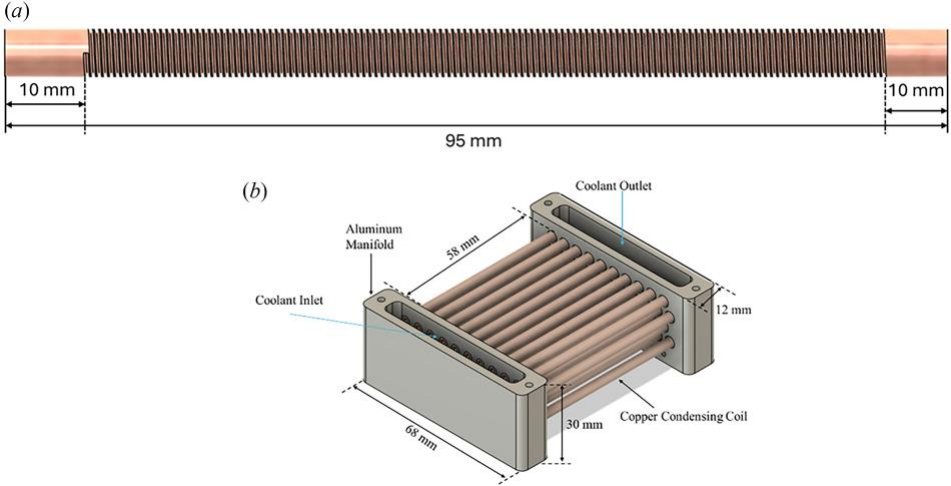
(*a*) Schematic of copper pipe for enhanced condenser and (*b*) isometric view of the condenser geometry with each respective dimensions

Two condenser designs were tested. As shown in Figs. 3(*a*) and 3(*b*), the bare condenser version employs 48 coils of 3.175 mm (1/8 in.) diameter copper tubing with a combined surface area of 26,225 mm^2^; this coil sits partially submerged in the working fluid to maximize heat rejection. The enhanced condenser version, as shown in Fig. 3(*a*), uses 28 finned tubes with a 4.75 mm outer diameter and 25 annular machined threads per inch; these tubes extended to a greater axial length, within the boiling chamber compared to the bare condenser design shown in Fig. 3(*a*), resulting in a total tube length of 95 mm, giving a total condensing area of 48,440 mm^2^. The difference in tube length between the two configurations arises from the distinct condenser geometries and mounting arrangements within the boiling chamber. Degassed, distilled water is used as the working fluid to minimize the influence of dissolved gases. Surface temperatures are measured using thermocouples inserted into small holes drilled into the copper heater block and substrates. The heater substrate is clamped firmly against the chamber base with 6–32 stainless–steel screws to seal the boiling surface within the chamber. The entire assembly bolts to a top and bottom aluminum plate to complete the test module.

**Fig. 4:**
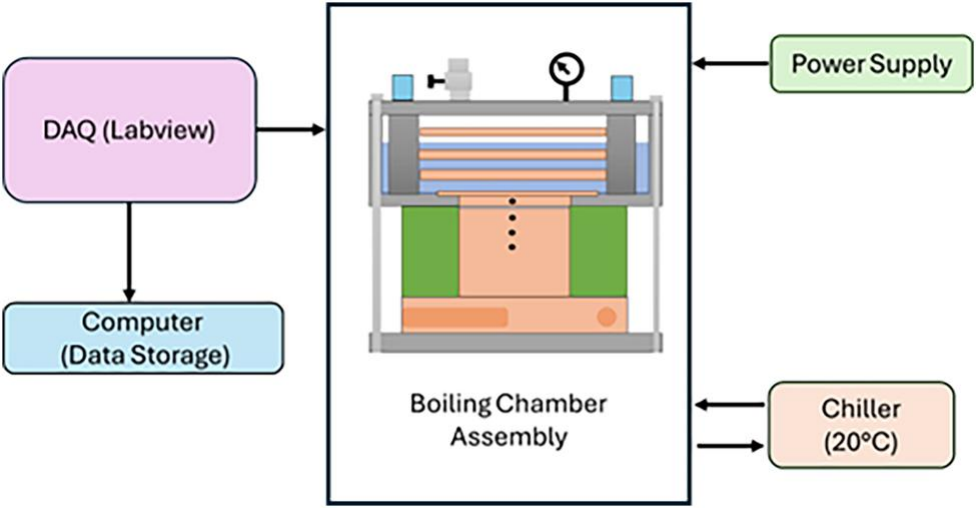
Flow diagram of the experimental setup, highlighting the integration of the chiller, power supply, data acquisition system (labview), and data storage components for precise temperature control and analysis. Adapted from Shukla and Kandlikar [11].

Data collection, as shown in Fig. 4, is handled using labview, which continuously records temperature, heat flux, and surface temperatures using calibrated K–type thermocouples. The instrumentation package includes a National Instruments cDAQ–9174 chassis equipped with NI–9214 modules for thermocouples and an National Instruments, Austin, TX (NI)–9205 module for analog inputs.

Thermocouples are positioned at critical points as shown in Table 1. labview from NI automates data acquisition and exports directly to Excel. Calibration is performed using an omega hot point cell from 50 °C to 200 °C in 25 °C steps; at each step, 50 readings are averaged. A linear correction derived from these calibrations is built into labview to eliminate system bias and uncertainties in the measured heat flux.

**Table 1:** Thermocouple locations for the experimental setup

Thermocouple	Location
T1	The top surface of the heater block
T2	Located in the middle section of the heater block
T3	The bottom surface of the heater block
T4	Located inside the substrate close to the surface
T5	It is in the chamber and will read the pool's boiling temperature.
Tin	Located on the inlet pipe
Tout	Located on the outlet pipe

### 3.3 Experimental Procedure.

Distilled water was introduced into the chamber at the prescribed fill ratio. After stabilizing the external chiller at the target coolant inlet temperature, a thin, uniform layer of thermal paste is applied to the copper heating substrate. This substrate was then precisely aligned with the heater block and secured firmly using stainless steel button-head screws. Finally, the inlet and outlet tubing connected the condensing coil to the chiller system. To improve measurement accuracy, dissolved gases were removed through vacuum degassing by heating the water. The saturation temperature was determined from the chamber's pressure gauge and standard steam tables. Reducing the chamber pressure to approximately 12 kPa lowered the water's saturation temperature to 49 °C, as determined from the measured absolute pressure using saturated water property tables, enabling nucleate boiling at reduced surface temperatures. Voltage increments of 5 V initiated the experimental sequence. The system reached steady-state conditions at each power level before advancing to the next increment.

### 3.4 Heat Transfer Calculation.

The heat flux (*q″*) represents the heat transferred per unit area from the bottom to the surface of the heating substrate and is determined using Fourier's 1D conduction equation:

(1) $$q^{\prime \prime }=-{k\;}_{Cu}\frac{dT}{dx}$$

where *k* is the thermal conductivity of copper 101 alloy (99.999% purity) with a value of 391 W/(m·K) and d*T*/d*x* represents the temperature gradient in the substrate. The distance between each thermocouple (T1–T3) is Δ*x*, and the temperature gradient is computed using Taylor's backward series expansion

(2) $$\frac{dT}{dx}=\frac{3T_{1}-4T_{2}+T_{3}}{2\Delta x}$$

Applying Fourier's law, the surface temperature of the heating substrate $\left(T_{\mathrm{surface}}\right)$ It is calculated as follows:

(3) $$T_{\mathrm{surface}}=T_{4}-q^{\prime \prime }(\frac{x_{s}}{k_{\mathrm{Cu}}})$$

Using the obtained temperatures, the heat transfer coefficient (*h*) is determined by

(4) $$h=\frac{q^{\prime \prime }}{T_{\mathrm{surface}}-T_{\mathrm{sat}}}$$

The total thermal resistance, represented by *R*, describes the effectiveness of a boiling chamber in dissipating heat. The temperature difference is needed to disperse 1 W of heat. Measured in K/W, thermal resistance can be calculated as follows:

(5) $$R=\frac{(T_{\mathrm{surface}}-T_{\mathrm{sat}})+(T_{\mathrm{sat}}-T_{\mathrm{coolant},\mathrm{mean}})\;}{Q}$$

The mean coolant temperature, $T_{\mathrm{coolant},\mathrm{mean}}$ is defined as the average of the coolant inlet and outlet temperatures

(6) $$T_{\mathrm{coolant},\;\mathrm{mean}}=\frac{T_{\mathrm{in}}+T_{\mathrm{out}}}{2}$$

where $T_{\mathrm{in}}$ and $T_{\mathrm{out}}$ are, respectively, the coolant temperatures at the condenser inlet and outlet.

### 3.5 Uncertainty Analysis.

Experimental measurements were subject to error affecting data accuracy, categorized as either bias or precision errors.

- Bias errors are from systematic deviations in instrumentation. These were minimized through device calibration to align measurements with actual values.
- Precision errors stem from random scatter in repeated measurements under identical conditions, which cannot be calibrated.

Primary bias uncertainties in this study originated from three key sources: The purity specification of the copper material, thermocouple measurement accuracy, and vernier caliper dimensional tolerances. To mitigate these systematic errors, thermocouples were calibrated, and instrument-specific correction factors were implemented in the data acquisition system. Despite these measures, residual uncertainty persisted and was quantified through formal uncertainty analysis—the total heat flux uncertainty (Eq. (9)) combined contributions from both bias and precision errors

(7) $$U_{q^{\prime \prime }}=\sqrt{\left(B_{q^{\prime \prime }}^{2}+P_{q^{\prime \prime }}^{2}\right)}$$

Uncertainty analysis incorporated key parameter variations:

- Thermal conductivity of copper
- Thermocouple temperature measurements (T1–T3)
- Thermocouple spacing (Δ*x*)

Measurement uncertainties were estimated using the standard deviation about the mean, yielding 95–98% confidence intervals. Sensitivity coefficients quantifying how each parameter variation affects the calculated heat flux were derived from partial derivatives of the heat flux equation. This method offers a comprehensive analysis of the measurement uncertainty by showing exactly how much each factor contributed to the total. The resulting sensitivity coefficients are incorporated into the overall bias uncertainty framework (Eq. (7)). Then, for every individual measurement point, as described by Kline and McClintock [20]. Equation (8) calculated the final bias uncertainty by propagating each parameter's uncertainty through its corresponding sensitivity coefficient and is shown in Table 2

(8) $${\;U}_{q\prime \prime }=\;\sqrt{{\left(\frac{\partial q^{\prime \prime }}{\partial K_{\mathrm{cu}}}\right)}^{2}\;U_{k_{\mathrm{cu}}}^{2}+{\left(\frac{\partial q^{\prime \prime }}{\partial x}\right)}^{2}U_{x}^{2}+{\left(\frac{\partial q^{\prime \prime }}{\partial T_{1}}\right)}^{2}U_{T_{1}\;}^{2}+{\left(\frac{\partial q^{\prime \prime }}{\partial T_{2}\;}\right)}^{2}U_{T_{2}}^{2}+{\left(\frac{\partial q^{\prime \prime }}{\partial T_{3}}\right)}^{2}U_{T_{3}}^{2}}$$

where

**Table 2:** Bias uncertainty of experimental equipment

Parameter	Value	Unit	Bias Uncertainty	% Uncertainty
$k_{\mathrm{cu}}$	391.00	$\frac{W}{m{}^{\circ}C}$	9.00	2.30%
Δx	3.00	mm	0.010	0.33%
*Flow rate*	4.75	kg/min	0.007	0.35%
$T_{1}$	Varies with temperature	° C	0.046	Varies with temperature
$T_{2}$	Varies with temperature	° C	0.045	Varies with temperature
$T_{3}$	Varies with temperature	° C	0.044	Varies with temperature

-
$k_{\mathrm{cu}}\;$is the thermal conductivity of copper, with uncertainty ${\;U}_{k\_\mathrm{cu}}$.
- x is the thickness of the copper layer, with uncertainty $U_{x}$.
-
$T_{1}$, $T_{2},\;T_{3}$ is the measured temperatures at different points, with uncertainties $U_{T_{1}},\;U_{T_{2}},\;U_{T_{3}}$.

The surface-temperature uncertainty analysis for the boiling chamber indicates a total uncertainty of ±1.057 °C over the experimental range. This uncertainty arises from the propagation of individual measurement errors in heat flux calculations. Key contributors include thermocouple uncertainties (±0.044–0.046 °C), variations in the thermal conductivity of copper alloy 101 (±2.30%), and dimensional tolerances of the copper heater block thermocouple holes (±0.33%). The uncertainty in our heat flux measurements is about ±2.136 W/cm^2^, which is roughly 3.28%. This level of uncertainty leads to only slight variations in surface temperature. Notably, the T4 thermocouple introduces the most uncertainty at ±0.046 °C. Fortunately, this is a very low percentage, accounting for less than 0.1% of the absolute operating temperatures. This suggests that our experimental setup is highly effective at reliably evaluating the thermal performance of both plain and microchannel substrate designs, even under varying fill ratios and coolant inlet temperatures.

## 4 Results

This section examines boiling chamber performance across operational parameters, specifically assessing how the plain and microchannel substrates impact critical heat flux (CHF) and overall thermal resistance. We evaluate these effects by varying fill ratios and inlet coolant temperatures for both bare and enhanced condenser surfaces.

### 4.1 Fill Ratios Utilizing Bare and Enhanced Condensers.

This section compares the boiling performance of both the bare and enhanced condensers with the plain and microchannel substrates, examining the effects of varying fill ratios of 40% and 80%, all under an inlet coolant temperature of 20 °C and a constant flowrate of 0.06 kg/s.

The fill ratio for each case is calculated using the following equation:

(9) $$\mathrm{fill}\;\mathrm{ratio}\;\%=\frac{V_{\mathrm{liquid}}}{V_{\mathrm{total}}}$$

where $V_{\mathrm{liquid}}$ is the liquid volume within the chamber (mm^3^) and $V_{\mathrm{total}}$ is the total available volume within the chamber (plain = 115,008 mm^3^, enhanced = 116,661 mm^3^).

The fill ratio represents the percentage fraction of the liquid region, which directly influences the system's capability for sustained subcooled boiling and efficient submerged condensation.

#### 4.1.1 Plain Substrate Performance and Discussion.

**Fig. 5:**
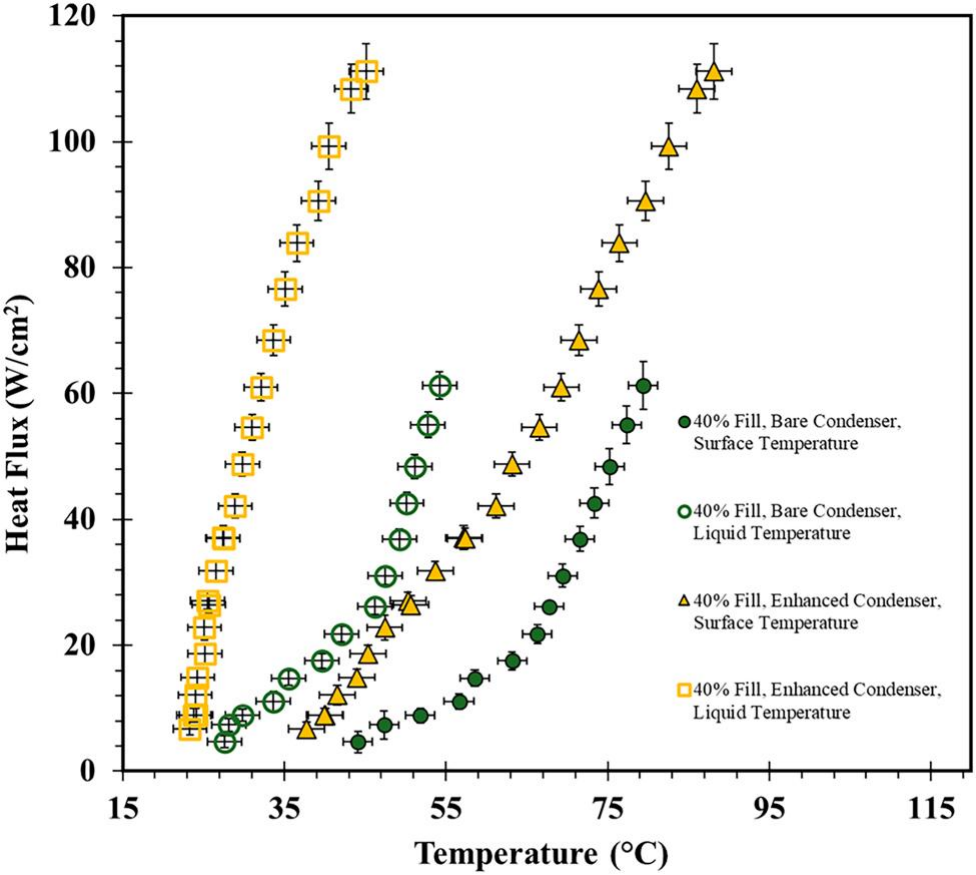
Comparison of the temperature heat flux for 40% fill ratio in the boiling chamber with plain substrate with the corresponding bulk liquid temperature shown

**Fig. 6:**
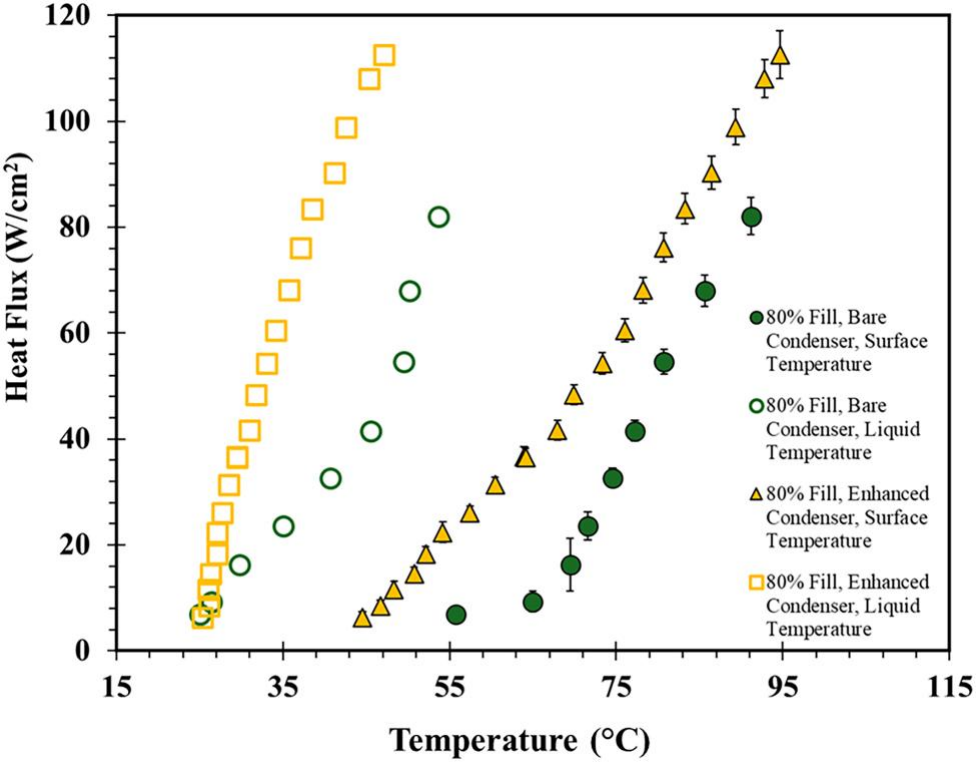
Comparison of the temperature heat flux for 80% fill ratio in the boiling chamber with the plain substrate with the corresponding bulk liquid temperature

This subsection evaluates the performance of the plain substrate at fill ratios 40% and 80% using both bare and enhanced condensers. Figures 5 and 6 show the performance of the boiling chamber with a bare condenser coil and with an enhanced condenser. The uncertainty of the heat flux and temperature readings is shown in Fig. 5, but is not fully displayed in later plots to avoid clutter. It is seen from these figures that the enhanced condenser achieves a CHF of approximately 112 W/cm^2^, which is approximately 1.8 times greater than the bare coil condenser. It is also seen that higher fill ratios tend to delay the onset of nucleate boiling. This delay results in elevated surface temperatures, which subsequently impair the system's overall performance. The plain substrate with a 40% fill ratio dissipated 110 W/cm^2^ at a surface temperature of 88 °C at CHF, when using the enhanced condenser. A fill ratio of 80% dissipated 115 W/cm^2^ with a surface temperature of 95 °C. In comparison, the bare condenser dissipated 60 W/cm^2^ at CHF with a surface temperature of 78 °C. Similarly, at 80% fill ratio with the bare condenser reached CHF of only 80 W/cm^2^, at a surface temperature of 90 °C.

It is observed that the enhanced condenser is able to maintain significantly lower bulk liquid temperatures. At 40% fill ratio, the enhanced condenser maintained the bulk liquid at 45 °C even at 110 W/cm^2^. In contrast, the bare condenser allowed the bulk liquid to reach 54 °C at only 61 W/cm^2^. This 9 °C reduction in temperature, despite nearly double the heat flux, directly translates into a higher degree of subcooling. The subcooling effect is defined as the temperature difference between the saturation temperature at system pressure and the actual bulk liquid temperature. By maintaining cooler bulk liquid conditions, the enhanced condenser preserves a larger driving temperature difference for heat transfer, which is fundamental to delaying CHF onset.

The increase in surface temperature as the fill ratio increases is mainly due to the rise in system pressure. This leads to an increase in the bulk liquid's saturation temperature; thus, a higher surface temperature is required to reap the benefits of nucleate boiling on the boiling surface. However, the enhanced condenser mitigates this effect through superior vapor condensation. At an 80% fill ratio, the enhanced condenser maintained a bulk liquid temperature of 47 °C, compared to 54 °C for the bare condenser, demonstrating consistent subcooling enhancement across fill ratios. This temperature suppression is crucial because subcooled pool boiling relies on the condensation of vapor bubbles as they rise through the cooler bulk liquid, and the enhanced condenser's ability to maintain lower liquid temperature enables sustained high-heat-flux operation.

A balance between the liquid and vapor regions is necessary to provide room for the vapor bubbles to detach and condense. The lower performance is attributed to the bare condenser tube design, characterized by a lower surface area and a higher pressure drop. This results in a higher mean coolant temperature through the condenser, which in turn leads to a higher bulk liquid temperature and an increase in pressure due to inadequate liquid replenishment. The enhanced condenser's increased surface area and improved flow characteristics enable more efficient vapor condensation, maintaining the subcooling necessary for stable nucleate boiling even at higher heat fluxes.

**Fig. 7:**
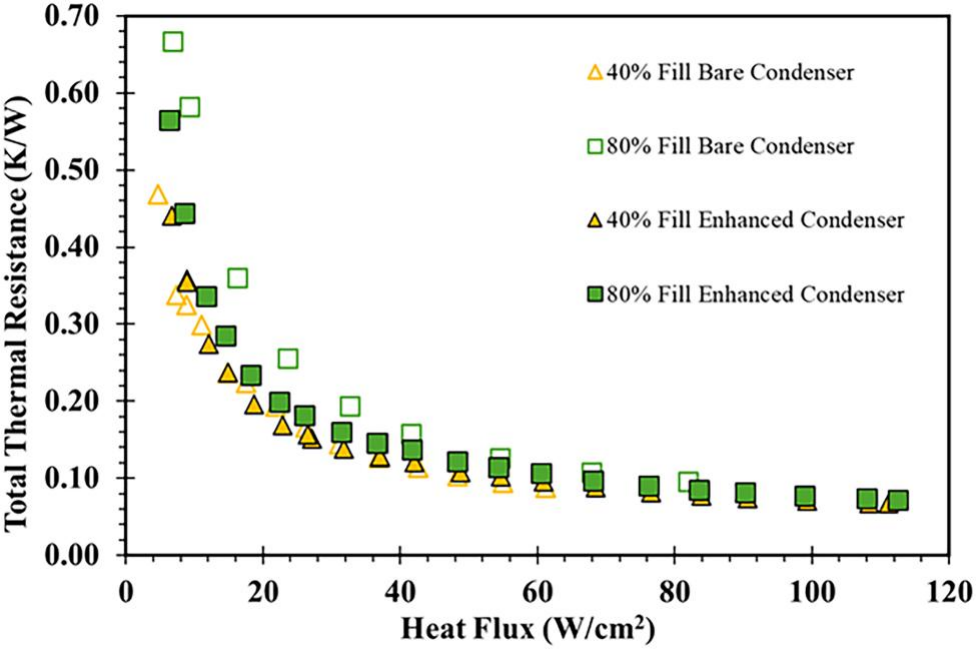
Variation of total thermal resistance as a function of heat flux for different fill ratios (40% and 80%) using both bare and enhanced condensers with an inlet coolant temperature of 20 °C

Figure 7 shows the comparison of the thermal resistance as heat is dissipated within the boiling chamber. The fill ratios for both the bare and enhanced condensers are shown, and a similar trend is observed where initially the thermal resistance decreases steeply as the heat flux increases. The higher fill ratios show a higher thermal resistance initially, but reach 0.05 K/W. At low heat fluxes, higher fill ratios result in higher initial thermal resistance because the increased liquid depth raises the pressure and delays the onset of nucleate boiling, making early heat transfer less efficient. The thermal resistance is high because heat is primarily transferred through natural convection or single-phase conduction from the boiling surface to the bulk liquid. In this regime, few or no vapor bubbles are generated; liquid motion is limited, and the thermal boundary layer at the surface is relatively thick. The lack of vigorous boiling means that the heat transfer coefficient is low, so a larger temperature difference between the surface and bulk liquid is required to remove a given amount of heat; thus, the system starts at a higher thermal resistance.

As the heat flux increases and the system transitions to nucleate boiling, numerous vapor bubbles form and depart from the heated surface. This boiling action disrupts the thermal boundary layer, which significantly increases the heat transfer coefficient. The result is that much more heat can be removed for a small increase in the surface temperature. Consequently, thermal resistance decreases dramatically with increasing heat flux. It converges, since nucleate boiling dominates and efficiently removes heat regardless of liquid level.

These findings demonstrate that, for the plain substrate, system-level improvements in condenser design are far more effective than changes in fill ratio for reducing overall thermal resistance and maximizing heat removal. Building on these results, the following subsection examines how microchannel surface enhancement further impacts boiling performance and thermal resistance under similar operating conditions.

#### 4.1.2 Microchannel Substrate Performance and Discussion.

**Fig. 8:**
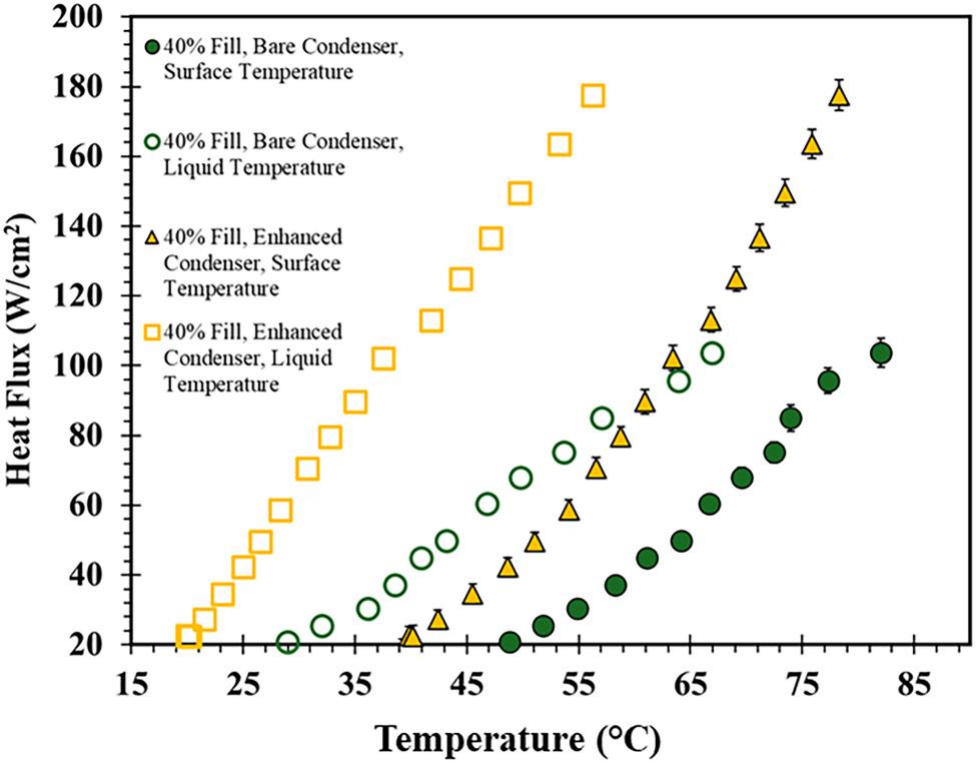
Comparison of the temperature heat flux for 40% fill ratio in the boiling chamber with the microchannel substrate, with the corresponding bulk liquid temperature shown

**Fig. 9:**
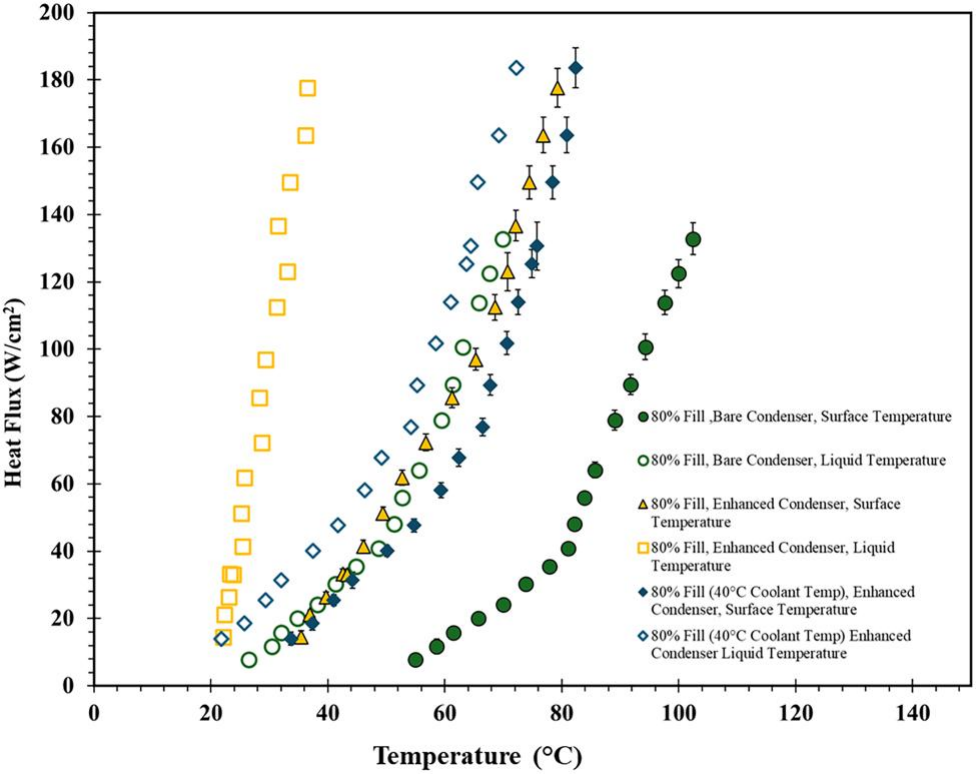
Comparison of the surface temperature heat flux for 80% fill ratio in the boiling chamber with microchannel substrate, with the corresponding bulk liquid temperature shown

This section examines the boiling and thermal resistance performance of the microchannel substrate with the bare and enhanced condensers, analyzing how different fill ratios affect system performance. Figures 8 and 9 present the boiling curves for both bare and enhanced condensers at various fill ratios. The results reveal a consistent trend, as the fill ratio increases, and the surface temperature increases slightly. This behavior mirrors that observed with the plain substrate configuration. The enhanced condenser significantly outperformed the bare condenser across all test conditions. While experiments with the enhanced condenser were limited by the maximum heat-supply capacity of the experimental setup (2000 W), the bare condenser reached CHF at lower heat fluxes. At a 40% fill ratio, the enhanced condenser achieved 178 W/cm^2^ with a surface temperature of 78 °C, while the bare condenser reached CHF at 103 W/cm^2^ with a surface temperature of 82 °C. This shows a dramatic improvement in performance, with the CHF almost double and the surface temperature lower. Similarly, at an 80% fill ratio, the enhanced condenser again achieved 177 W/cm^2^ with a surface temperature of 79 °C, using a 20 °C inlet coolant at CHF. 80% fill ratio with an inlet coolant temperature of 40 °C dissipated 183 W/cm^2^ with a surface temperature of 82 °C. In contrast, the bare condenser operated at 132 W/cm^2^ with a surface temperature of 102 °C, without reaching CHF.

The temperature behavior of the bulk liquid, illustrated in Figs. 8 and 9 reveal substantial differences between condenser configurations. For the 40% fill ratio shown in Fig. 8, the bare condenser exhibits a steady increase in temperature from approximately 20 °C to 67 °C at CHF at 103 W/cm^2^. In contrast, the enhanced condenser maintains significantly lower temperatures, reaching only 55 °C at 178 W/cm^2^, resulting in a 12 °C reduction and delaying CHF in comparison to the bare condenser. The 80% fill ratio in Fig. 9 demonstrates a similar pattern. As heat flux increases, the bare condenser shows a rapid temperature rise from 25 °C to approximately 70 °C, reaching CHF at 132 W/cm^2^. In comparison, the enhanced condenser maintains the bulk liquid temperature at just 36 °C at 178 W/cm^2^ without reaching CHF with a notable 24 °C temperature reduction. The microchannel substrate consistently outperformed the plain substrate across all fill ratios tested. Table 3 summarizes the maximum heat flux and corresponding surface temperatures for both substrate configurations with each condenser type. These results demonstrate the critical importance of enhanced condenser geometry for achieving efficient subcooled pool boiling, notably by maintaining lower bulk liquid temperatures and extending the operational range before CHF.

**Table 3:** Maximum heat flux and surface temperature for plain and microchannel substrates under different fill ratios and condenser types

Substrate type	Condenser	Fill ratio (%)	Max heat flux (W/cm^2^)	Surface temperature at max flux (°C)	Thermal resistance (K/W)
Plain	Bare	40	61^*^	79	0.086
Plain	Bare	80	82^*^	91	0.075
Plain	Enhanced	40	108^*^	88	0.049
Plain	Enhanced	80	109^*^	95	0.056
Microchannel	Bare	40	10^*^	82	0.051
Microchannel	Bare	80	132	102	0.052
Microchannel	Enhanced	40	178^*^	78	0.023
Microchannel	Enhanced	80	177^*^	79	0.023
Microchannel	Enhanced	80	183	82	0.017

Asterisk (*) indicates that CHF was reached.

**Fig. 10:**
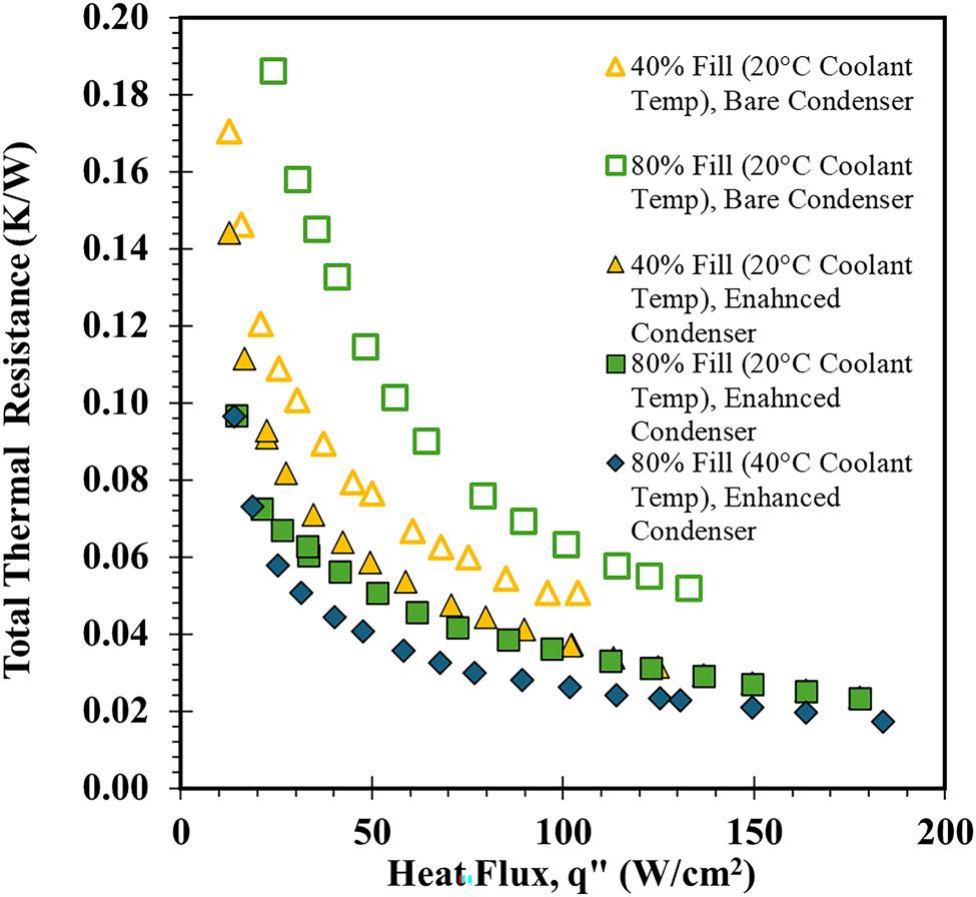
Total thermal resistance of the boiling chamber as a function of heat flux for 40%, and 80% fill ratios for the microchannel substrate

Figure 10 below shows the total thermal resistance for both condensers utilizing the microchannel substrate. It showed a total thermal resistance that is significantly lower when compared to the plain substrate. The initial thermal resistance for higher fill ratios is higher for both the bare and enhanced condensers. The enhanced condenser, however, had a lower initial thermal resistance in comparison to the bare condenser. It is observed that the total thermal resistance for the enhanced condenser at an 80% fill ratio with an inlet coolant temperature of 40 °C is 0.017 K/W at the maximum heat flux. The microchannel substrate with the enhanced condenser exceeds the most advanced cooling mechanisms as discussed in literature. In comparison, the plain substrate across all fill ratios was 0.05 K/W, highlighting that the total thermal resistance decreased due to the substrate microstructure. This trend is consistent for each full ratio initially which is shown to initially be higher at lower heat fluxes due to the rise in pressure due to vapor buildup within the boiling chamber.

It is essential to adjust both the amount of liquid and the shape of the condenser to reduce heat resistance and improve cooling efficiency in the boiling chamber. While enhancements in the surface microstructures and an optimal fill ratio can lower thermal resistance during nucleate boiling, the overall system performance is ultimately governed by condenser design, which dictates the efficiency of vapor condensation and liquid replenishment at elevated heat loads. Having established the roles of fill ratio and condenser enhancement in governing boiling and thermal resistance characteristics, the subsequent section will focus on the influence of coolant inlet temperature, offering further insight into the coupled effects of system operating parameters on high heat flux thermal management while also eliminating the need for a cooling tower.

### 4.2 Coolant Temperatures.

The following subsections discuss the boiling chamber under different inlet coolant temperatures of 20 °C, 30 °C, 40 °C, and 50 °C with a 40% fill ratio and a flowrate of 0.06 kg/s which is the maximum value that can be attained with the current system. The results are compared with the plain and microchannel substrates for both the bare and enhanced condensers, as well as the total thermal resistance is also analyzed. The coolant inlet temperature is a crucial operating parameter where a coolant inlet temperature of 40 °C can eliminate the need for a cooling tower and enable air cooling, thus significantly reducing water and energy usage in data centers.

#### 4.2.1 Plain Substrate Performance and Discussion.

**Fig. 11:**
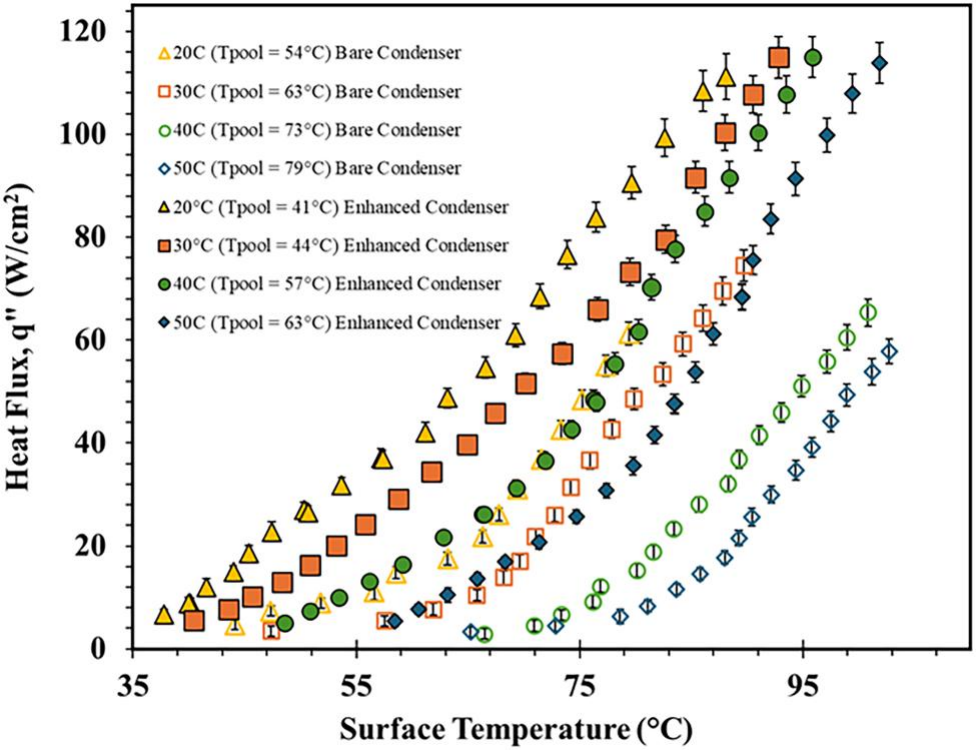
Comparison of surface temperature while dissipating heat for the plain substrate under varying coolant inlet temperatures (20 °C, 30 °C, 40 °C, and 50 °C) for both bare and enhanced condensers

Figure 11 presents the boiling curve for the plain substrate under varying coolant inlet temperatures (20 °C, 30 °C, 40 °C, and 50 °C) for both the bare and enhanced condensers. For each configuration, increasing the coolant inlet temperature results in a systematic shift of the boiling curve toward higher surface temperatures at a given heat flux. In the case of the bare condenser, a coolant temperature of 20 °C with a bulk liquid temperature of 54 °C achieves the lowest surface temperatures of 79 °C while dissipating 61 W/cm^2^ while the 50 °C inlet which had a bulk liquid temperature of 79 °C at 60 W/cm^2^ shows the highest surface temperature of 102 °C, reflecting a reduced temperature gradient for condensation and less effective vapor removal. A similar trend is observed for the enhanced condenser, though the absolute surface temperatures remain substantially lower across all coolant conditions. The plain substrate dissipated 108 W/cm^2^ at a surface temperature of 88 °C for an inlet coolant temperature of 20 °C, while at an inlet coolant temperature of 50 °C, the surface temperature was 99 °C. The enhanced condenser showed substantial improvements where the inlet coolant temperature of 50 °C outperformed the bare condenser with an inlet coolant temperature of 30 °C. The enhanced condenser did not reach CHF while the bare condenser did at the maximum heat flux.

**Fig. 12:**
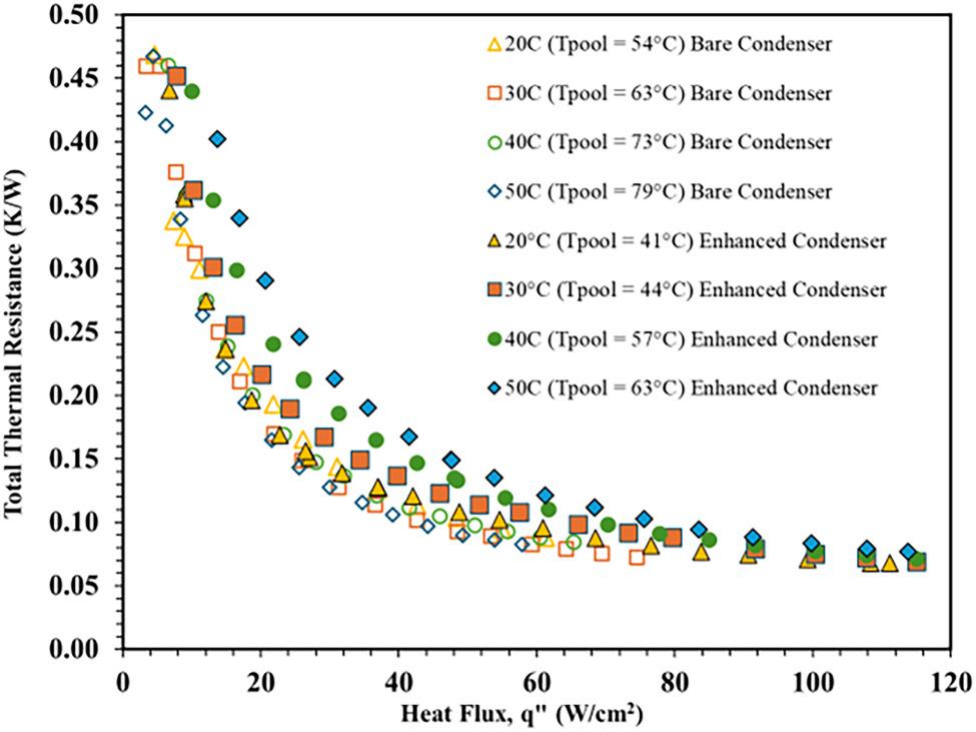
Comparison of total thermal resistance while dissipating heat for bare and enhanced condensers with the plain substrate at coolant inlet temperatures from 20 °C to 50 °C

Figure 12 presents the total thermal resistance as a function of heat flux for both bare and enhanced condensers across coolant inlet temperatures of 20 °C, 30 °C, 40 °C, and 50 °C. For both the bare and enhanced condenser designs, the thermal resistance decreases exponentially with increasing heat flux and converges to 0.05 K/W at maximum heat flux, regardless of the coolant inlet temperature. The initial thermal resistance is higher at elevated coolant temperatures for both bare and enhanced condensers due to the reduced temperature difference between the bulk liquid temperature and the boiling surface, which delays the onset of vigorous nucleate boiling and condensation.

These results for the plain substrate demonstrate how both coolant inlet temperature and condenser enhancement govern boiling heat transfer, particularly at low and moderate heat fluxes where thermal resistances are most sensitive to boundary conditions. At high heat flux, performance differences for the thermal resistance diminish, but the enhanced condenser consistently shows lower overall resistance and improved stability across all coolant temperatures. The following section extends this analysis to the microchannel substrate, enabling direct comparison of thermal behavior and performance under elevated coolant temperature conditions.

#### 4.2.2 Microchannel Substrate Performance and Discussion.

**Fig. 13:**
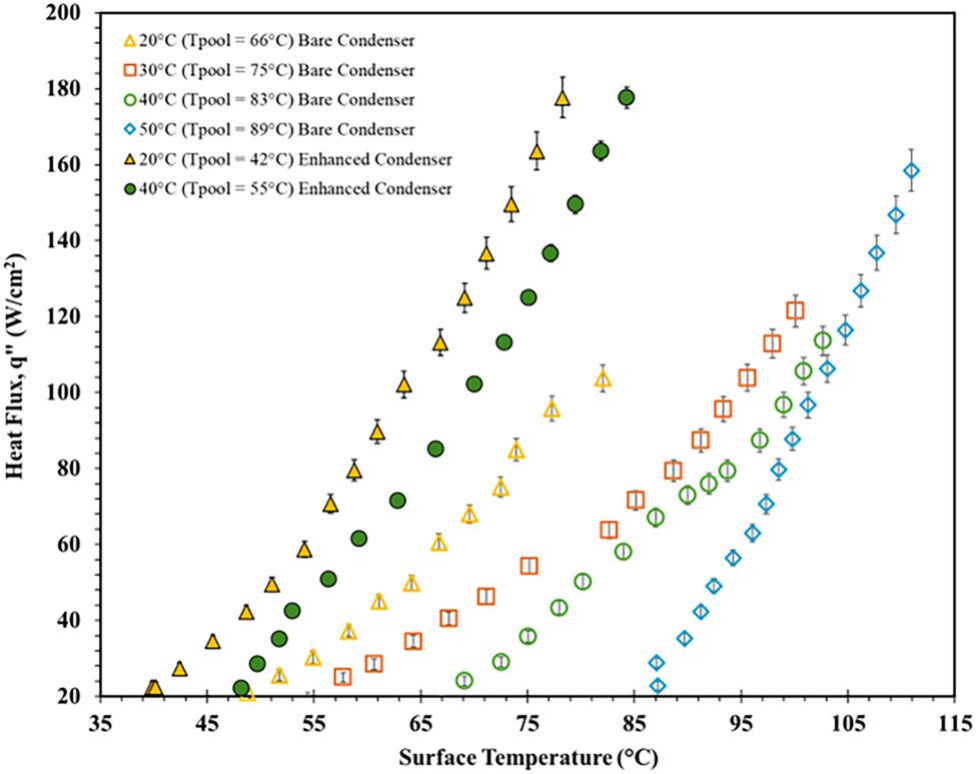
Comparison of the microchannel substrate under varying coolant inlet temperatures (20 °C, 30 °C, 40 °C, and 50 °C) with bare and enhanced condensers

The following subsection presents a detailed analysis of the boiling performance for the microchannel substrate under varying coolant inlet temperatures (20 °C, 30 °C, 40 °C, and 50 °C), using both bare and enhanced condensers. Figure 13 shows the comparison between the surface temperature while dissipating heat. Across all coolant temperatures, the microchannel substrate demonstrates a substantial enhancement in heat dissipation compared to the plain substrate, reaching higher maximum heat fluxes at lower surface temperatures.

For the bare condenser, CHF occurred only at 20 °C, 30 °C, and 40 °C inlet coolant temperatures. The maximum heat fluxes for these cases were approximately 102 W/cm^2^ at 20 °C, 121 W/cm^2^ at 30 °C, and 113 W/cm^2^ at 40 °C, all at surface temperatures of 82 °C, 100 °C, and 102 °C, respectively. At 50 °C coolant inlet, the bare condenser did not reach CHF within the tested range, but still achieved a high heat flux of 158 W/cm^2^, with the surface temperature approaching 110 °C.

In contrast, the enhanced condenser enabled the microchannel substrate to sustain even greater heat fluxes without reaching CHF, especially as the coolant inlet temperature increased. At a 20 °C inlet coolant temperature, the maximum heat flux was approximately 178 W/cm^2^, with a surface temperature of 78 °C. At 40 °C inlet temperatures, the enhanced condenser maintains heat fluxes up to 177 W/cm^2^ at 84 °C without reaching CHF. The results demonstrate that a microchannel substrate paired with an improved condenser supports high heat flux dissipation at lower surface temperatures and delays or prevents CHF onset, even as the coolant inlet temperature rises. This combination is highly effective at sustaining elevated cooling performance, eliminating the need for evaporative cooling in data centers.

**Fig. 14:**
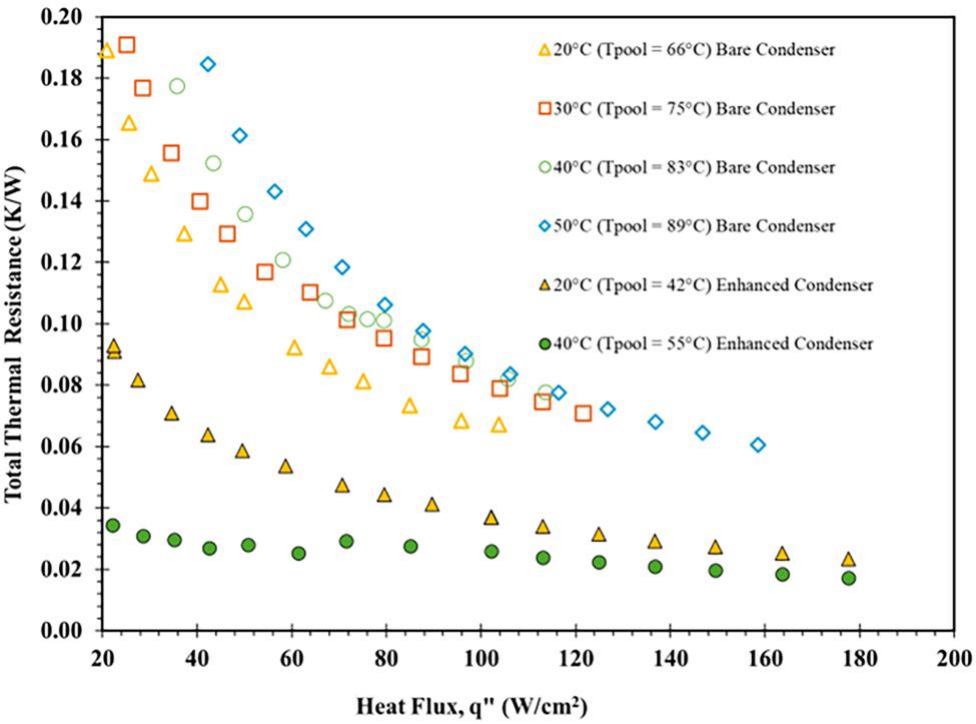
Total thermal resistance as a function of heat flux for the microchannel substrate under various coolant inlet temperatures (20 °C to 50 °C) and condenser types

Figure 14 illustrates the total thermal resistance as a function of heat flux for the microchannel substrate under different coolant inlet temperatures (20 °C, 30 °C, 40 °C, and 50 °C) and for both bare and enhanced condensers. As heat flux increases, thermal resistance decreases sharply for all bare-condenser cases, converging to approximately 0.06 K/W at the highest heat flux, regardless of coolant temperature. The enhanced condenser, however, consistently achieves lower thermal resistance at both 20 °C and 40 °C, with values of 0.021 K/W and 0.018 K/W, respectively. This shows how the thermal resistance decreases even at an elevated coolant temperature of 40 °C. This not only outperforms the most advanced cooling mechanisms but also does so at elevated coolant inlet temperatures, eliminating the need for evaporative cooling and potentially significantly reducing water and energy use.

These results demonstrate that combining microchannel substrates with enhanced condenser design within the boiling chamber maintains low thermal resistance even at higher coolant temperatures. This approach offers a promising solution to meet the growing demands of next-generation electronic devices in data centers.

The following section will put these results into context within the broader landscape of high-performance computing hardware and emerging cooling methods. A detailed comparison is provided against commercial CPU and GPU thermal design limits as well as innovative two-phase and immersion cooling technologies reported in recent literature, underscoring both the practical implications and future opportunities for next-generation data center thermal management.

#### 4.2.3 Comparison of Cooling Methods.

**Fig. 15:**
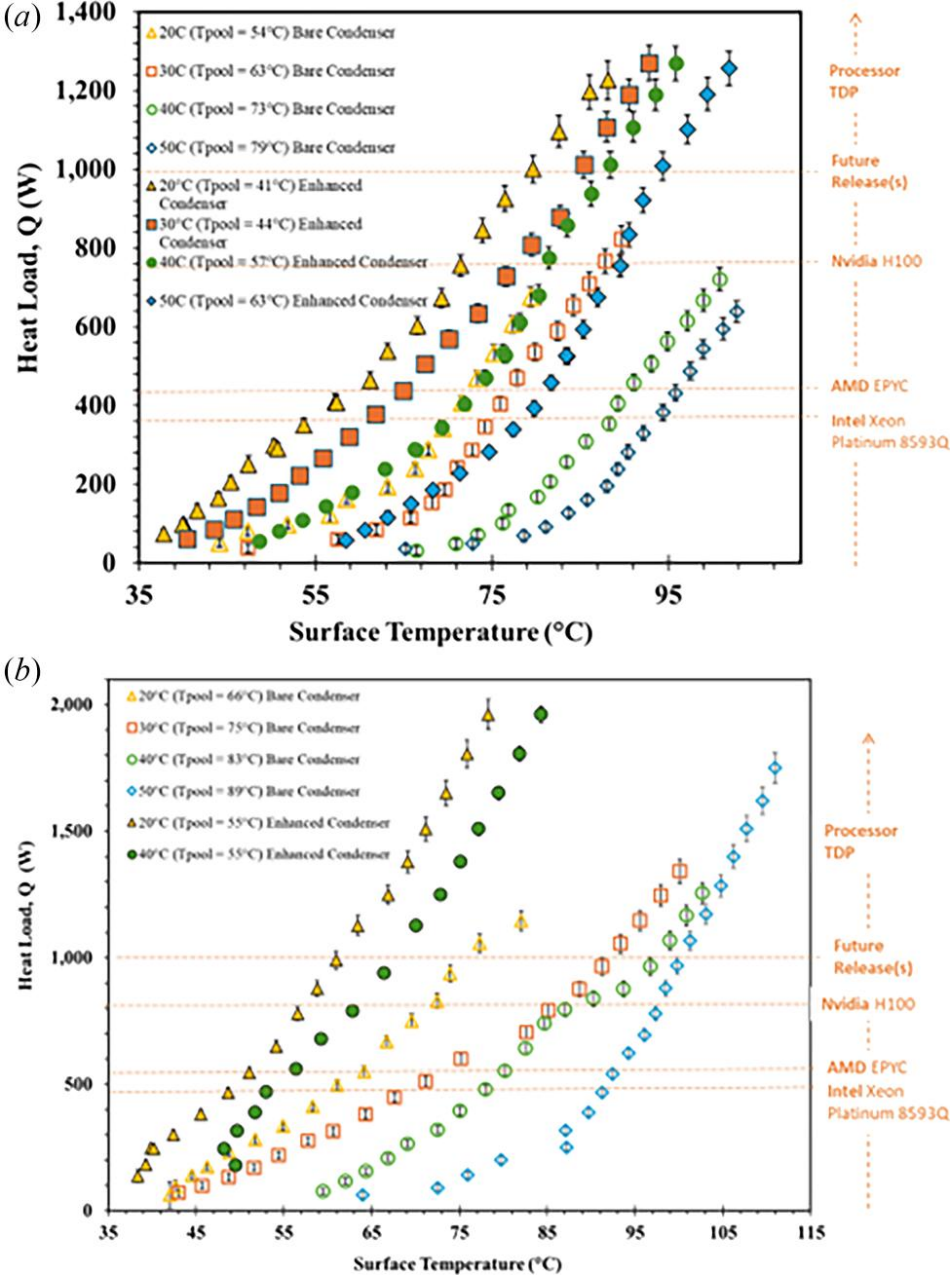
(*a*) comparison of the surface temperature while dissipating heat for the plain substrate and (*b*) for the microchannel substrate, under coolant inlet temperatures of 20 °C–50 °C with bare and enhanced condensers

Figure 15 compares the heat load versus surface temperature for the plain substrate (*a*) and microchannel substrate (*b*) across coolant inlet temperatures from 20 °C to 50 °C, with overlayed reference lines indicating the thermal design power of major CPUs and GPUs including the Intel Xeon Platinum 8593Q (∼350 W), advanced micro devices (AMD) EPYC (∼400 W), Nvidia H100 (∼700 W), and projections for future processor releases above 1000 W [21–29]. For the plain substrate, the enhanced condenser at a 50 °C coolant inlet achieves heat loads up to approximately 900 W at surface temperatures below 90 °C, meeting or exceeding the requirements for current data center CPUs and GPUs. In contrast, the microchannel substrate in Fig. 13(*b*) demonstrates even greater heat dissipation: at a 40 °C coolant inlet, the enhanced condenser enables sustained heat loads exceeding 1900 W at surface temperatures under 85 °C. These results confirm that, even at higher coolant temperatures suitable for air cooling, both substrate configurations, especially the microchannel substrate, can exceed the thermal requirements of today's highest-performance processors and future models.

For context, as detailed in Table 1 in Sec. 1, existing commercial and research solutions such as vapor chambers, thermosyphons, and immersion cooling typically support maximum CPU heat loads up to 900 W per device [20–25]. The boiling chamber approach presented here not only surpasses these values, but does so with a compact, direct-contact architecture targeting the primary sources of heat (CPUs and GPUs). Having efficient heat dissipation even at elevated coolant inlet temperatures, this innovative system enables air-cooled operation, significantly lowering energy consumption and minimizing water usage. Moreover, it eliminates reliance on conventional cooling towers, paving the way for scalable, environmentally sustainable cooling solutions for next-generation data centers.

Table 4 summarizes the maximum heat load and corresponding surface temperature for a range of representative cooling methods, including air cooling, water cooling, a dual-taper thermosyphon loop, and the boiling chamber system tested with both microchannel and plain substrate configurations at a 20 °C coolant inlet. Traditional air coolers support a maximum heat load of 110 W at a surface temperature of 64 °C, while conventional water coolers and thermosyphon loops achieve 255 W and 268 W at 66 °C and 63 °C, respectively [17]. In stark contrast, the boiling chamber system demonstrates a dramatic enhancement in cooling performance: the microchannel substrate configuration sustains a heat load of 1544 W at a surface temperature of 83 °C, and the plain substrate achieves 1196 W at 88 °C. These results underscore the significant heat removal advantage of the boiling chamber, particularly when paired with microchannel enhancements, far surpassing the capabilities of traditional and advanced single- and two-phase cooling technologies under the same boundary conditions.

**Table 4:** Comparison of maximum heat load and corresponding surface temperature for leading cooling methods and boiling chamber configurations at 20 °C coolant inlet

Cooling method	Max heat load (W)	Surface temperature (°C)
Air cooler [11]	110	64
Water cooler [11]	255	66
Dual-taper thermosyphon loop [11]	268	63
Boiling chamber with enhanced condenser and plain substrate at 20 °C	1196	88
Boiling chamber with enhanced condenser and microchannel substrate at 20 °C	1950	78

## 5 Conclusion

In this study, we evaluated the thermal performance of a novel boiling chamber utilizing the bare and enhanced condenser surfaces with varying fill ratios and inlet coolant temperatures for high heat flux dissipation. The main findings are as follows:

1. Enhanced condenser surfaces substantially reduced total thermal resistance compared to bare designs, especially at higher fill ratios and coolant inlet temperatures. The lowest total thermal resistance was 0.017 K/W with the microchannel substrate and the enhanced finned tube configuration at an inlet coolant temperature of 40 °C, outperforming other cooling mechanisms.
2. Microchannel substrate geometry enabled significant improvements in boiling heat transfer. The microchannel substrate, in combination with the enhanced condenser, achieved maximum heat loads up to 1.95 kW at a surface temperature of 78 °C and 20 °C coolant inlet. At a 40 °C coolant inlet, the system sustained 178 W/cm^2^ (1.95 kW) at surface temperatures below 80 °C.
3. Critical heat flux was either delayed or not reached within experimental limits for the enhanced condenser, indicating a robust margin for thermal safety at practical data center operating conditions.
4. The 80% fill ratio with an inlet coolant temperature of 40 °C dissipated 183 W/cm^2^ at a surface temperature of 82 °C without reaching CHF. The total thermal resistance was 0.017 K/W. The experiments were stopped to avoid damage to the experimental setup.
5. Performance comparison with existing cooling methods, including air, water, thermosyphon, and immersion cooling showed that the boiling chamber far surpassed the heat load limits of these technologies. Traditional solutions supported 110–300 W per device, whereas the boiling chamber supported over 1900 W with the microchannel substrate.
6. High coolant inlet temperature operation was demonstrated without significant loss in performance, supporting the use of air-cooled condensers and enabling the elimination of cooling towers. This has direct implications for energy and water savings in large-scale data centers.
7. Compact chamber design targets the main heat sources (CPUs and GPUs) directly, offering a scalable approach compatible with the thermal requirements of current and future processor generations.

## Nomenclature

*A*_submerged_ =: submerged condensing coil area, mm^2^
*A*_total_ =: total condensing coil surface area, mm^2^
*B* =: bias error
CHF =: critical heat flux, W/cm^2^
*dT*/*dx* =: temperature gradient, °C/m
FRC =: fill ratio coefficient, dimensionless
*h* =: heat transfer coefficient, W/(m^2^·K)
*k* =: thermal conductivity, W/(m·K)
*P* =: precision error
PUE =: power usage effectiveness, dimensionless
*q″* =: heat flux, W/cm^2^
*R* =: total thermal resistance, K/W
$T_{\mathrm{coolant},\;\mathrm{mean}\;}$ =: mean coolant temperature, °C
*T*_in_, *T*_out_ =: coolant inlet/outlet temperature, °C
$T_{\mathrm{sat}}$ =: saturation temperature of fluid, °C
$T_{\mathrm{surface}}$ =: surface temperature of substrate, °C
T1 =: temperature at the top of the heater block, °C
T2 =: temperature at the middle of the heater block, °C
T3 =: temperature at the bottom of the heater block, °C
T4 =: temperature inside the substrate near the surface, °C
T4 =: temperature inside the substrate near the surface, °C
T5 =: pool boiling temperature, °C
$U_{\mathrm{total}}$ =: total uncertainty, dimensionless
*x* =: thickness of the copper layer, cm
Δ*T* =: temperature difference, °C
Δ*x* =: distance between thermocouples, cm
*σ* =: standard deviation, dimensionless
